# Mesh-augmented transvaginal repair of recurrent or complex anterior pelvic organ prolapse in accordance with the SCENIHR opinion

**DOI:** 10.1007/s00192-020-04525-9

**Published:** 2020-09-24

**Authors:** Gert Naumann, Tanja Hüsch, Claudia Mörgeli, Anna Kolterer, Ralf Tunn

**Affiliations:** 1 Department of Gynecology and Obstetrics, Helios Hospital Erfurt, Nordhäuser Straße 74, 99089 Erfurt, Germany; 2 Department of Gynecology and Obstetrics, University Hospital Düsseldorf, Düsseldorf, Germany; 3 Department of Urology and Pediatric Urology, University Medical Center of Johannes Gutenberg University, Mainz, Germany; 4 Promedon GmbH, Clinical Research, Kolbermoor, Germany; 5 Department of Urogynecology, St. Hedwig Hospital, Berlin, Germany

**Keywords:** Cystocele, Pelvic floor disorders, Pelvic organ prolapse, Surgical mesh, Surgery, Therapy

## Abstract

**Introduction and hypothesis:**

To evaluate the performance of mesh-augmented repair of anterior pelvic organ prolapse (POP) with or without apical vaginal wall involvement in women with recurrent or complex prolapse.

**Methods:**

This multicenter cohort study included women undergoing surgery with Calistar S (Promedon, Argentina) for anterior POP between 2016 and 2018. The SCENIHR opinion was considered for patient selection, surgeon’s experience and choice of implant. Patients were prospectively invited to assess effectiveness and safety by anamnesis, validated questionnaires and pelvic examination. A composite endpoint defined by POP-Q ≤ 1, absence of a vaginal bulge symptom and repeated surgery for POP was used to define treatment success. Descriptive statistics were applied. McNemar or Wilcoxon signed rank tests were used as paired samples tests. The significance level was set at 5%.

**Results:**

A total of 107 non-fertile women with a mean age of 70.6 years were enrolled. Ninety-three (86.9%) women presented with recurrent prolapse. The mean follow-up time was 18.5 months. Treatment success was achieved in 76% of cases according the composite endpoint, with 98% reaching POP-Q ≤ 1 and a significant improvement in quality of life (*p* < 0.001). Mesh exposure occurred in six (5.6%) patients, although none required further surgery. Four (3.7%) patients reported dyspareunia, and a single (0.9%) patient displayed a prominence due to mesh folding.

**Conclusions:**

Mesh-augmented repair of anterior POP is effective and safe in women with recurrent or complex prolapse. Hence, in a select patient population, the benefits of mesh-augmented POP repair still outweigh the risks.

**Electronic supplementary material:**

The online version of this article (10.1007/s00192-020-04525-9) contains supplementary material, which is available to authorized users.

## Introduction

The utilization of synthetic implants in pelvic organ prolapse (POP) repair has become increasingly popular in the last decade because of the high failure rate of native tissue repair [1, 2]. There is convincing evidence that synthetic mesh is superior to native tissue repair in the anterior compartment [3]. However, there have been patient safety concerns regarding meshes used for POP repair [1]. A number of observed adverse events were described as difficult to manage or were associated with a risk of permanent morbidity [4]. Since then, in addition to surgeon experience and enhanced patient selection, progress in the development of meshes, including changed composition, improved design, and reduced mesh densities, have been established as factors that significantly reduce the rates of these adverse events [1].

Historically, the risk-benefit ratio for transvaginal mesh-augmented repair has been critically reviewed by various urogynecology associations and by the Scientific Committee on Emerging and Newly Identified Health Risks (SCENIHR). The SCENIHR is one of several scientific committees established by the European Commission to provide a comprehensive independent assessment of risks to consumer safety or public health and related issues. The committee gives independent scientific advice and risk assessment for the development and monitoring of Union policies and legislation related to public health and consumer safety of the European Commission with the goal of improving public health and protecting citizens and the environment [5]. Most associations agree that in a select patient population, the benefits of transvaginal mesh repair still outweigh the risks [3, 6]. The SCENIHR concluded that mesh-augmented repair should be limited to women with recurrent or complex POP [3], whereby complex POP refers to primary prolapse with high risk of POP recurrence, e.g., other surgical procedures are expected to fail in these women [3]. However, there is only one study investigating the performance of mesh-augmented POP repair in this patient population. Marschke et al. [7] evaluated the performance of Elevate Anterior (Astora Women’s Health, Eden Prairie, MN, USA) for anterior POP repair in women with recurrent or complex POP, demonstrating satisfactory success rates and few adverse events. However, Elevate Anterior has been removed from the worldwide market. Calistar S provides a comparable implantation technique and mesh design and therefore was subsequently utilized in the participating study centers.

Calistar S (Promedon, Cordoba, Argentina) is a biocompatible, type 1 (monofilamentous, macroporous), ultra-lightweight polypropylene mesh used for the treatment of anterior POP with or without apical vaginal wall involvement. The mesh is implanted in a single-incision transvaginal fashion with single-use instruments provided in the mesh kit. Importantly, Calistar S fulfills the recent recommendations regarding the compounds and design for mesh-augmented POP repair [3, 8]. The novel ultra-lightweight design of the mesh aims to further reduce mesh-related complications [9]. Calistar S has been available since 2015, and approximately 15,000 kits have been sold so far. There is one retrospective trial comparing its predecessor, Calistar A, with Calistar S [10]. The composite endpoint for surgical success was reached in 90% of the patients with Calistar S and, according to validated questionnaires, QoL improved significantly relative to baseline. Mesh exposure or extrusion occurred in five (4%) patients.

The current investigation aims to evaluate the performance of mesh-augmented repair in women with anterior POP in a select patient population with mid-term follow-up, while considering the recommendations by the SCENIHR opinion on the safety of surgical meshes used in urogynecological surgery [3], i.e., patient selection, surgeon experience and choice of implant. Accordingly, the population is composed of women with recurrent or primary complex anterior prolapse. Calistar S was selected for this study as it was already established in the participating centers, being routinely used by experts in pelvic floor reconstruction via vaginal route. Calistar S fulfills the proposed requirements for implants in transvaginal POP repair according to the SCENIHR recommendations. The article was prepared in accordance with the STROBE reporting checklist.

## Materials and methods

This was a multicenter single-arm cohort study that was approved by the local ethics committees of the participating centers (60,750/2019/99). Each patient provided written informed consent for participation in this investigation. Women who had been operated on with Calistar S for anterior pelvic organ prolapse with or without apical vaginal wall involvement between March 2016 and December 2018 in the study sites were invited to participate in this clinical investigation. Women who voluntarily agreed for participation in this trial were included. Exclusion criteria included missing data for the retrospective data collection as well as refusal or inability to participate. The number of participants reflects the total number of surgeries performed in both study centers and the total number of patients who provided consent for the trial. All surgeons implanting Calistar S were experienced in transvaginal pelvic floor reconstruction. The indication for mesh-augmented POP repair in both study centers conformed with the SCENIHR opinion; thus, only women with recurrent symptomatic prolapse or primary symptomatic complex POP with high risk of recurrence, i.e., other surgical procedures were expected to fail [3], were considered for mesh-augmented repair. Additionally, only non-fertile women were considered for a mesh implant.

Patient data on demographic information and perioperative course were collected retrospectively according the medical records. The validated *German Pelvic Organ Prolapse Questionnaire* (GPOP-Q) was routinely employed in the study centers prior to surgical treatment. The GPOP-Q is a validated, standardized quality of life (QoL) questionnaire for women with POP, including four domains (bowel, urinary, sexual and prolapse symptoms) with scoring ranging from 0 to 10, whereas a higher score indicates a more negative impact as well as a total score (range 0–40) combining the results of all domains [11]. Pain was assessed at follow-up using the visual analog scale (VAS) (range 0–10, a higher score indicates a more negative impact). To assess treatment success and complication rates, patients were invited prospectively (at time of enrollment) to a clinical appointment, at least 6 months after the operation, where the medical history was updated and vaginal examination was performed. Furthermore, the patients prospectively completed the VAS for pain and the GPOP-Q.

Treatment success was defined by a composite endpoint including anatomical and subjective components as well as the necessity for a repeated surgery due to POP recurrence [12], as follows:

1. A POP-Q stage of ≤ 1 for the leading edge of the anterior or apical vaginal wall,
2. Absence of a vaginal bulge symptom and
3. No need for repeated surgery for anterior or apical POP.

Anatomical success was defined by POP-Q stage of ≤ 1 (Ba and C < −1 cm). Complication rates are presented according to the Clavien-Dindo classification.

The following variables were utilized to identify risk factors for complications: surgeon, body mass index, POP-Q stage, smoking status, number of deliveries, prior urogynecological surgeries, diabetes mellitus status, age, study site, pre- and/or postoperative estrogen supplementation and concurrent hysterectomy.

### Statistics

Descriptive data are presented as the median [min, max] and mean ± standard deviation values, as appropriate. Categorical variables are presented as numbers and frequencies. Time-dependent variables are presented using Kaplan-Meier curves. Differences between groups were tested using the Mann-Whitney U test, Fisher’s exact test or log-rank test, as appropriate. McNemar or Wilcoxon signed rank tests were used as paired samples tests. Data missing from the current data set were handled via an available case analysis approach, and sensitivity analysis was not performed. Quantitative variables were not grouped. The significance level was set at 5%.

### Surgical technique

A hydrodissection for developing the vesicovaginal space followed by a vaginal midline incision and full thickness vaginal wall dissection is performed. Then, the rectovaginal and subsequently pararectal space is developed by blunt and sharp dissection, as appropriate. The ischial spines and sacrospinous ligaments are identified by palpation. The surrounding tissue of the sacrospinous ligament is wiped away carefully from the ischial spine along the ligament using the index finger. The tissue anchoring system (TAS) has single anchors attached with non-absorbable monofilamentous sutures. The anchor is fixed to the sacrospinous ligament (SSL) by single-use instruments bilaterally. Subsequently, dissection towards the obturator foramen horizontally to the bladder neck is performed. The anchors of the ventral part of the implant are attached to the mesh [so-called anterior attachment arms (AAAs)]. The AAAs are fixed into the bulge of the internal obturator muscle by introducing the AAA parallel to the obturator membrane with single-use instruments. The central part of the mesh is then attached by two absorbable sutures close to the bladder neck to prevent displacement. The posterior central part of the mesh is attached with two non-absorbable sutures to the pericervical ring or, in case of hysterectomy, to the remnants of the cardinal ligaments. Subsequently, the posterior mesh arms are fixed to the SSL by knotting the corresponding sutures of the TAS. Wound closure according to the surgeon’s preference and vaginal packing. A schematic diagram of the implantation technique is demonstrated in Fig. S1.

### Sponsor role

This clinical investigation was funded and sponsored by Promedon GmbH. The study design, data interpretation and decision for publication were developed in collaboration between the investigator and sponsor. Due to the retrospective character of this trial, the indication of mesh-augmented repair was independently evaluated by the investigators and corresponded to the SCENIHR opinion. Conduction of the trial and data collection were performed by the investigators. Data management was provided by the sponsor. Data analysis was performed by an independent statistician. The manuscript was written by the sponsor based on the agreement about the content and extent of the manuscript by the authors. Editing and final approval of the manuscript was required of each author. Importantly, the investigators had the ultimate authority over each activity.

## Results

A total of 107 women with a mean age of 70.6 (SD 7.67) years were enrolled (Figure S2). The mean follow-up time was 18.5 (SD 8.02) months. Most patients presented with recurrent anterior POP [*n* = 93 (86.9%)] and were postmenopausal [*n* = 105 (98.1%)]. Two women who were not postmenopausal had undergone prior hysterectomy. The median POP-Q stage was 3 at baseline. The complete list of baseline characteristics is presented in Table 1.

**Table 1:** Baseline characteristics

Variable		*n* = 107
Age in years, mean (SD)		70.6 (7.67)
Primary complex vs. recurrent POP, *n* (%)	Primary complex	14 (13.1)
	Recurrent	93 (86.9)
Body mass index (kg/m^2^), mean (SD)		27.8 (3.92)
Smoking status, *n* (%)	Never smoker	57 (53.3)
	History of smoking	3 (2.8)
	Current smoker	2 (1.9)
	Missing data	46 (43.0)
Diabetes mellitus, *n* (%)		16 (14.9)
Asthma or COPD, *n* (%)		6 (5.6)
Residual urine volume in ml, mean (SD)		79.5 (99.7)
Menopausal status, *n* (%)	Postmenopausal	105 (98.1)
	Pre- or perimenopausal	2 (1.9)
Number of child births, *n* (%)	0	4 (3.7)
	1	24 (22.4)
	2	52 (48.6)
	3	17 (15.9)
	≥ 4	10 (9,3)
Stress urinary incontinence, *n* (%)		31 (29.0)
Prior urogynecological surgeries, *n* (%)	History of hysterectomy	84 (78.5)
	History of anterior colporrhaphy	86 (80.4)
	History of posterior colporrhaphy	18 (16.8)
	History of sacrocolpopexy	9 (8.4)
	History of sacrospinous fixation by the Amreich-Richter technique	15 (14.0)
	History of mesh-augmented POP repair or midurethral sling	8 (7.5)
POP-Q staging		
Anterior vaginal wall, *n* (%)	2	23 (21.5)
	3	81 (75.7)
	4	3 (2.8)
Apical vaginal wall, *n* (%)	0	44 (41.1)
	1	9 (8.4)
	2	13 (12.1)
	3	32 (29.9)
	4	8 (7.5)
	Missing data	1 (0.9)
Posterior vaginal wall, *n* (%)	0	39 (36.4)
	1	51 (47.6)
	2	13 (12.1)
	3	3 (2.8)
	Missing data	1 (0.9)

POP: pelvic organ prolapse, POP-Q: Pelvic Organ Prolapse Quantification, COPD: chronic obstructive pulmonary disease

Anatomical success was accomplished in 98 (98.0%, Table 2) patients. Treatment success according to the primary composite endpoint was achieved in 76 (76.0%) patients. The POP-Q stage was not documented adequately in seven (6.5%) patients. However, each of these seven patients was satisfied or very satisfied with the procedure, and prolapse symptoms improved in all patients. Furthermore, none of these patients required repeated surgery for POP. Anatomical failure occurred in two patients, as evidenced by POP-Q ≥ 2, though repeated surgery was not indicated for either of these patients by the time of last follow-up. One of these patients reported no prolapse symptoms, and the other patient experienced only infrequent prolapse symptoms. The estimated anatomical failure-free rate was 97% after 16.7 months (Figure S3).

**Table 2:** POP-Q staging and composite primary endpoint assessment at follow-up

Compartment	POP-Q stage	*n* = 100
Anterior vaginal wall, *n* (%)	0	85 (85.0)
	1	13 (13.0)
	2	2 (2.0)
	3 or 4	0
Apical vaginal wall, *n* (%)	0	96 (96.0)
	1	4 (4.0)
	2, 3 or 4	0
Posterior vaginal wall, *n* (%)	0	44 (44.0)
	1	31 (31.0)
	2	19 (19.0)
	3	6 (6.0)
	4	0
Composite endpoint		
Leading edge of the anterior and apical vaginal wall POP-Q ≤ 1, *n* (%)		98 (98.0)
Absence of the vaginal bulge symptom, *n* (%)		77 (77)
No need for repeated surgery for anterior or apical POP,* n* (%)		100 (100)
Success according to the primary composite endpoint, *n* (%)		76 (76%)

POP-Q: Pelvic Organ Prolapse Quantification, POP: pelvic organ prolapse

**Fig. 1:**
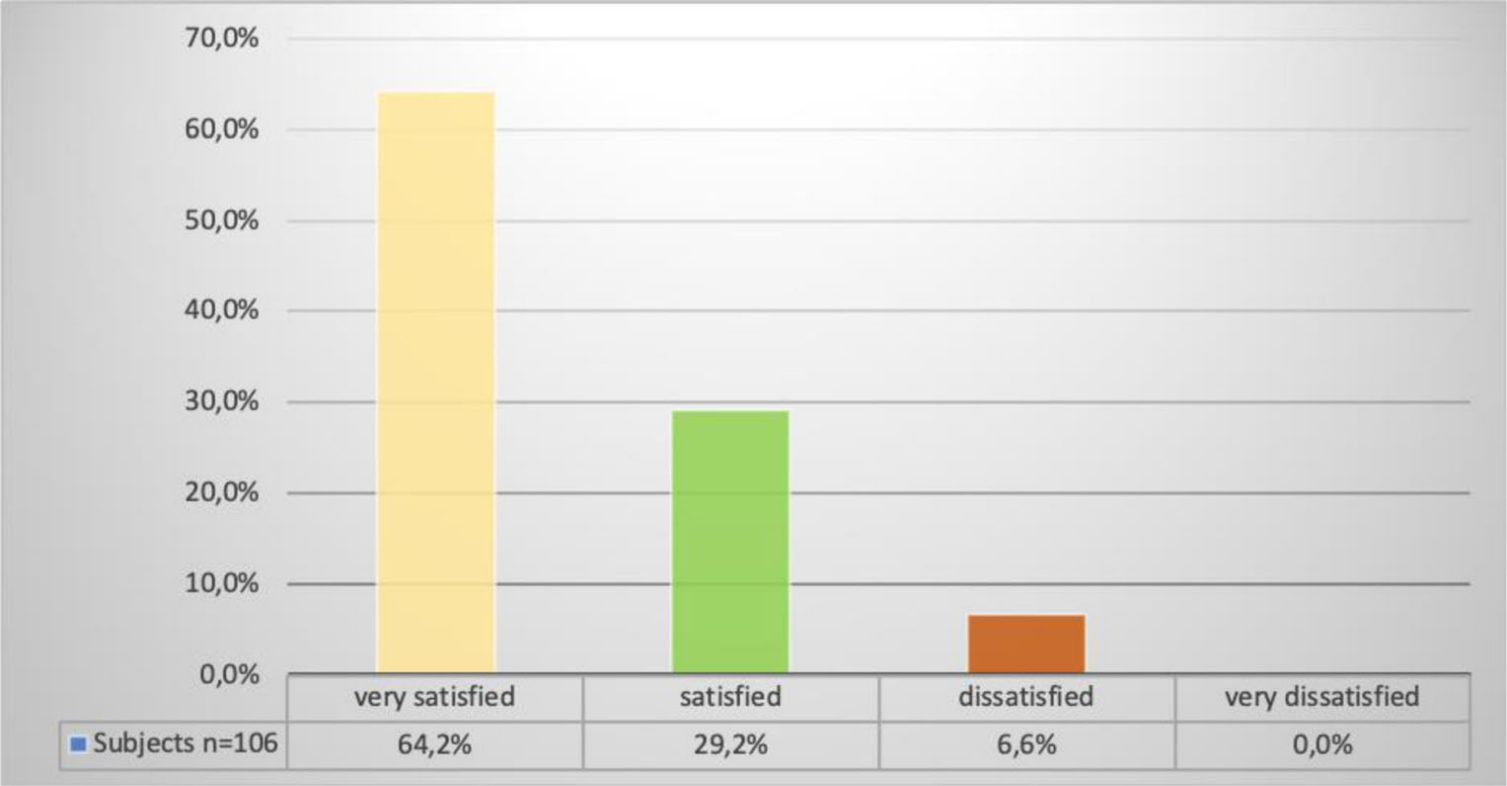
Patient satisfaction with the operation

A total of 99 of 106 (93.4%) patients were either satisfied or very satisfied with the surgery. Seven (6.6%) were dissatisfied with the procedure (Fig. 1). Six patients reported not being satisfied because of a persistent or de novo bladder dysfunction or urinary incontinence, and one patient reported dissatisfaction because of pain.

**Fig. 2:**
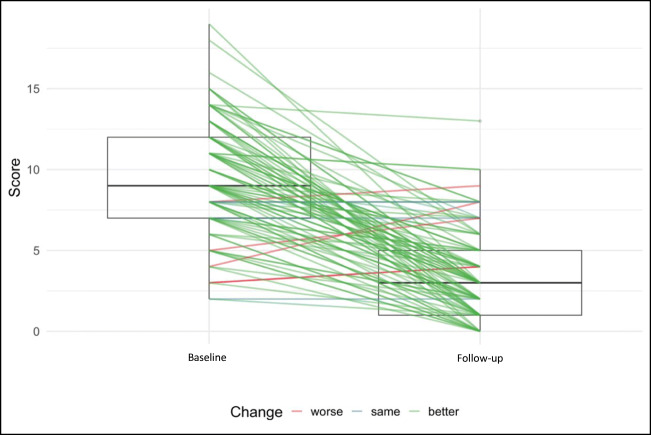
Change in total score of the German Pelvic Organ Prolapse-Questionnaire comparing baseline to follow-up results

Prolapse-related symptoms and QoL improved significantly in each of the GPOP-Q domains compared to baseline (Table 3). The total score of the GPOP-Q decreased significantly by 6.4 (*p* < 0.001, Fig. 2). Further results are presented in the supplementary material (Figure S4 and S5).

**Table 3:** Change in domain scores of the German Pelvic Organ Prolapse Questionnaire comparing baseline to follow-up

Domain	Baseline	Follow-up	Mean difference	*p* value
	(*n* = 107)	(*n* = 107)		
Bladder function, mean (SD) [n (%)]	3.29 (1.84) [99 (93.4)]	1.97 (1.63) [100 (93.5)]	- 1.32	< 0.0001*
Bowel function, mean (SD) [n (%)]	1.65 (1.34) [92 (86.0)]	1.37 (1.36) [97 (90.7)]	- 0.28	< 0.0001*
Prolapse symptoms, mean (SD) [n (%)]	5.16 (2.69) [91 (85.8)]	0.33 (0.65) [97 (90.7)]	- 4.83	0.005*
Sexual function, mean (SD) [n (%)]	1.91 (2.32) [99 (93.4)]	0.88 (1.4) [100 (93.5)]	- 1.03	< 0.0001*
Total score, mean (SD)	10.4 (1.97)	4.04 (1.75)	- 6.36	< 0.0001*

*Significant: *p* < 0.05; SD standard deviation

The mean duration of surgery was 37.7 (SD 17.3) min. No intraoperative complications occurred, in particular, no organ, vessel or nerve injury or blood loss of > 200 ml. During the postoperative course, one (0.9%) patient developed an urinary tract infection and was treated with antibiotics. Mesh exposure occurred in six (5.6%) patients, all of which were < 1 cm in diameter. Exposures required no treatment or could be managed with vaginal estrogen therapy. In one (0.9%) patient, an exposed suture was cut during an ambulatory vaginal examination. None of the erosions required further surgery at the time of last follow-up. The estimated exposure-free rate was 93% after 20 months (Figure S6). Mesh exposure was not associated with any of the defined risk factors. The complete list of complication rates, classified according to the Clavien-Dindo scale, is presented in Table 4.

**Table 4:** Complication rates classified according the Clavien-Dindo scale

Variable	Clavien-Dindo grading *n* = 107						
	NA	I	II		III		IV
			a	b	a	b	
Bacterial or mycotic vaginosis, *n* (%)			2 (1.9)				
Clinical infection of the study device, *n* (%)	0						
Impaired wound healing, *n* (%)			1 (0.9)				
Exposure, *n* (%)		6 (5.6)					
Symptomatic residual urine or urinary retention, *n* (%)		4 (3.7)					
Constipation, *n* (%)		10 (9.3)					
Pain according to VAS, mean (SD)		0.131 (0.688)					
Dyspareunia, *n* (%)		4 (3.7)					
De novo stress urinary incontinence or worsening of urinary incontinence, *n* (%)	11 (10.3)**					9 (8.4)*	
Contraction of the study device, *n* (%)	0						
Dehiscence, *n* (%)	0						
Folding of the mesh, *n* (%)						1 (0.9)	
Rectocele/enterocele, *n* (%)						2 (1.9)^†^	

NA, not applicable

*Elective surgery for midurethral sling or bulking agent for the treatment of stress urinary incontinence

**No treatment due to patient refusal and/or lack of symptoms

^†^Elective surgery for symptomatic prolapse in posterior compartment

One patient (0.9%) required further surgery because of folding without epithelial separation. Trimming of the mesh was performed during concomitant implantation of a retropubic midurethral sling. Four (3.7%) women had persistent residual urine or urinary retention that required intermittent catheterization.

Furthermore, 66 of 98 (67.3%) women reported not participating in any regular sexual activity, and 19 (19.4%) reported infrequent sexual activity. A total of 13 (13.3%) subjects were regularly sexually active. Of four (4.1%) patients with dyspareunia, three (3.1%) reported seldom and one (1.0%) no sexual activity. Furthermore, dyspareunia was present prior to the operation in three (3.1%) of these women.

## Discussion

Mesh-augmented anterior POP repair with Calistar S demonstrated a favorable risk/benefit ratio in non-fertile women with recurrent POP or primary complex POP with high risk of recurrence. Treatment success was 76.0% according to the composite endpoint and 98.0% according to the anatomical criteria POP-Q ≤ 1. No intraoperative complications occurred, no patient required repeated surgery for POP, and QoL increased significantly after the intervention. Importantly, postoperative complications were rare, and none of the patients that developed mesh exposure required further surgery. Further surgery for stress urinary incontinence was performed in nine (8.4%) patients. Due to the inclusion of women with recurrent and primary complex POP, in accordance with the SCENIHR recommendations [3], the current investigation reflects valuable results from clinical daily practice in the use of mesh-augmented transvaginal repair of anterior POP with Calistar S.

The anatomical success rate was consistent with previously reported success rates for mesh-augmented anterior POP repair, which range between 83 and 100% with a follow-up time of up to 36 months [13–17]. Despite the strict definition of no subjective vaginal bulge symptom in the composite endpoint, success was still achieved in 76% of the patients. Although a composite endpoint is still rarely utilized in reporting POP repair outcomes, there are several recommendations for this strategy, which includes anatomical and subjective components as well as the need for retreatment [12]. Unsurprisingly, success rates decrease if a composite endpoint is utilized [18]. Previous studies in mesh-augmented anterior POP repair report success rates between 60.8 and 87.0% when utilizing a composite endpoint [2, 14, 16], so that our results are in line with previous publications.

In contrast to previous reports of mesh-augmented repair, it is important to note that the vast majority of our patients presented with recurrent or complex POP instead of a primary POP, so that the overall complexity of our cohort was considerably higher, despite similar success rates.

Two subjects in this investigation experienced objective failure, as defined by POP-Q grade 2. However, neither of these patients required an additional surgery for POP up to the follow-up, reporting no or only infrequent prolapse symptoms.

Regarding the intraoperative course, the mean duration of surgery was 37.7 minutes, which is consistent with other reports in the literature and significantly faster than native tissue repairs or other repair procedures [2]. Intraoperative complications are generally rare in transvaginal mesh repair, and the most frequent complication is bladder injury, with a reported incidence of between 1 and 4 % [2, 19]. No intraoperative complications occurred in this investigation.

Vaginal mesh exposure occurred in 5.6% of the patients in our study, and the estimated exposure-free rate was 93% after 20 months. Since none of our patients required further surgery up to the follow-up, all exposures could be managed conservatively. The reported exposure rates in the literature range between 3.2 and 14% [2, 13–17, 20–25]. Exposure rates have decreased successively in the last decade because of stricter patient selection, advancements in mesh compounds and design and improvement of surgical techniques. Importantly, exposure rarely requires further surgery [2, 13–17, 20–24]. Our results are consistent with the literature, despite the relatively high mean age of our patients (70 years), which is associated with reduced estrogen levels and an increased risk for vaginal atrophy [19], which ultimately increases the risk for mesh exposure.

A single patient required further surgery because of folding/wrinkling. In this patient, the mesh was trimmed during an elective suburethral sling implantation to treat stress urinary incontinence. Nonetheless, folding and wrinkling are known complications that can occur after mesh implantation for POP [26]. Mesh folding is usually identified by imaging methods alone. Nevertheless, the implication of this finding is still unclear. There are contradictory reports that mesh folding is associated with an increased risk for exposure, and it remains unclear whether this results from mesh contraction or weak fixation points. To reduce this complication, the anchors of Calistar S were developed to resist a force exceeding four times the maximum abdominal pressure.

Due to persistent postoperative bladder dysfunction, 3.7% of patients required intermittent catherization. This pre-existing dysfunction was observed at baseline and is likely due to neuronal or structural tissue damage and not caused by the surgical POP repair. Consistent with the literature, de novo stress urinary incontinence occurred in 10.2% and worsening of urinary incontinence in 8.3% of patients [19]. These results could be explained by the pathophysiology of the disease, as occult urinary stress incontinence is commonly present in POP patients, particularly those with higher degrees of POP [27]. Bladder function improved in the majority of patients in our investigation.

Pain levels were low with a mean VAS score of 0.13. Dyspareunia was reported by 4.1% of cases, although this condition was already documented in 3.1% of patients at baseline. Therefore, only one (1.0%) patient reported de novo dyspareunia after the operation. Dyspareunia is reported to occur in between 2.7% and 10% of patients in the literature [15, 20, 23], which is consistent with our results. It is important to note that the number of patients participating in regular sexual activity was only 13.3% in our cohort. The mean age of patients in the current trial was 70 years, and sexual activity may be reduced by lack of libido, physical limitations or lack of a partner [28]; consequently, the impact of sexual impairment will differ compared with other populations.

We acknowledge potential bias due to the funding source of this trial. However, the investigators had the ultimate authority over each activity in this clinical investigation. Due to their focus on urogynecology, the participating hospitals have documented baseline characteristics consistently and accurately for years, as reflected by the low amount of missing data. Nevertheless, we acknowledge limitations in the study design affecting the amount and quality of baseline data. Bias due to different perioperative assessments and surgeons cannot be completely excluded, although results are consistent with those in the literature, and complications were rare. All involved institutions were experienced in the treatment of transvaginal POP repair and therefore represented the current standard of care. Finally, the current trial provides only mid-term data. Despite these limitations, the results reflect current routine clinical practice and provide valuable information regarding the performance of Calistar S in the intended population.

In conclusion, considering the SCENIHR recommendations for a suitable mesh implant, adequate patient selection and surgeon experience in transvaginal pelvic floor reconstruction, our study demonstrates that mesh-augmented transvaginal repair with Calistar S is an effective and safe option in women with recurrent or primary complex anterior compartment prolapse. Although the majority of our patients had recurrent prolapses, success rates remained high and re-intervention rates for recurrence or adverse events were deniable in this select population.

## Electronic supplementary material

ESM 1 (DOC 104 kb)

ESM 2 (DOCX 216 kb)

ESM 3 (DOCX 24.2 kb)

ESM 4 (DOCX 107 kb)

ESM 5 (DOCX 319 kb)

ESM 6 (DOCX 336 kb)

ESM 7 (DOCX 99.1 kb)
