# Supplementary material for: Mesh-augmented transvaginal repair of recurrent or complex anterior pelvic organ prolapse in accordance with the SCENIHR opinion
Source: Int Urogynecol J. 2020 Sep 24;32(4):819–27. doi: 10.1007/s00192-020-04525-9 (PMC8009781; doi:10.1007/s00192-020-04525-9)
Supplement: Supplementary file 1 — (DOC 104 kb) [file 192_2020_4525_MOESM1_ESM.doc]

STROBE Statement—Checklist of items that should be included in reports of ***cohort studies***

**Mesh-augmented transvaginal repair of recurrent or complex anterior pelvic organ prolapse in accordance with SCENIHR-opinion**

|  | Item No | Recommendation | Reported on Page Number & Line Number | Reported on Section & Paragraph |
| --- | --- | --- | --- | --- |
| **Title and abstract** | 1 | (*a*) Indicate the study’s design with a commonly used term in the title or the abstract | page 3, line 39 | Abstract, Paragraph 2 |
|  |  | (*b*) Provide in the abstract an informative and balanced summary of what was done and what was found | page 3, line39 - 53 | Abstract, Paragraph 2-3 |
| Introduction | | |  |  |
| Background/rationale | 2 | Explain the scientific background and rationale for the investigation being reported | page 5, line 65-100 | Introduction, Paragraph 1-3 |
| Objectives | 3 | State specific objectives, including any prespecified hypotheses | page 5, line 102-111 | Introduction, Paragraph 4 |
| Methods | | |  |  |
| Study design | 4 | Present key elements of study design early in the paper | page 7, line 113-125 | Methods, Paragraph 1 |
| Setting | 5 | Describe the setting, locations, and relevant dates, including periods of recruitment, exposure, follow-up, and data collection | page 7, line 115-118, line 127-138 | Methods, Paragraph 1 and 2 |
| Participants | 6 | (*a*) Give the eligibility criteria, and the sources and methods of selection of participants. Describe methods of follow-up | page 7, line 115-124, line 134-137 | Methods, Paragraph 1 and 2 |
|  |  | (*b*) For matched studies, give matching criteria and number of exposed and unexposed | *not applicable* | *not applicable* |
| Variables | 7 | Clearly define all outcomes, exposures, predictors, potential confounders, and effect modifiers. Give diagnostic criteria, if applicable | page 7-8, 127-152 | Methods, Paragraph 2-4 |
| Data sources/ measurement | 8* | For each variable of interest, give sources of data and details of methods of assessment (measurement). Describe comparability of assessment methods if there is more than one group | page 7, line 127-130, 135-138 | Methods, Paragraph 2 |
| Bias | 9 | Describe any efforts to address potential sources of bias | page 8, line 149-152 | Methods, Paragraph 4 |
| Study size | 10 | Explain how the study size was arrived at | page 7, 124-125 | Methods, Paragraph 1 |
| Quantitative variables | 11 | Explain how quantitative variables were handled in the analyses. If applicable, describe which groupings were chosen and why | page 8, line 160 | Methods, Paragraph Statistics |
| Statistical methods | 12 | (*a*) Describe all statistical methods, including those used to control for confounding | page 8, line 154-161 | Methods, Paragraph Statistics |
|  |  | (*b*) Describe any methods used to examine subgroups and interactions | page 8, line 156-157 | Methods, Paragraph Statistics |
|  |  | (*c*) Explain how missing data were addressed | page 8, line 158-159 | Methods, Paragraph Statistics |
|  |  | (*d*) If applicable, explain how loss to follow-up was addressed | not applicable | not applicable |
|  |  | (*e*) Describe any sensitivity analyses | page 8, line 159-160 | Methods, Paragraph Statistics |
| Results | | |  |  |
| Participants | 13* | (a) Report numbers of individuals at each stage of study—eg numbers potentially eligible, examined for eligibility, confirmed eligible, included in the study, completing follow-up, and analysed | page 9, line 181, Figure S1 | Results, Paragraph 1 and Figure S1 |
|  |  | (b) Give reasons for non-participation at each stage | page 8, line 181, Figure S1 | Results, Paragraph 1 Reference to Figure S1 |
|  |  | (c) Consider use of a flow diagram | Supplementary material | Figure S1 |
| Descriptive data | 14* | (a) Give characteristics of study participants (eg demographic, clinical, social) and information on exposures and potential confounders | page 9, line 182-185, Table 1 | Results, Paragraph 1; Table 1 |
|  |  | (b) Indicate number of participants with missing data for each variable of interest | Table 1-4 | Table 1-4 |
|  |  | (c) Summarise follow-up time (eg, average and total amount) | page 9, line 182 | Results, Paragraph 1 |
| Outcome data | 15* | Report numbers of outcome events or summary measures over time | Table 2-4, page 9-10, line 187-228 | Table 2-4, Results Paragraph 2-7 |
| Main results | 16 | (*a*) Give unadjusted estimates and, if applicable, confounder-adjusted estimates and their precision (eg, 95% confidence interval). Make clear which confounders were adjusted for and why they were included | page 11 line 215-217 | Results Paragraph 5, Results Paragraph 5 |
|  |  | (*b*) Report category boundaries when continuous variables were categorized | not applicable | not applicable |
|  |  | (*c*) If relevant, consider translating estimates of relative risk into absolute risk for a meaningful time period | not applicable | not applicable |
| Other analyses | 17 | Report other analyses done—eg analyses of subgroups and interactions, and sensitivity analyses | page 10 line 195-196, page 11 line 214-215 | Results Paragraph 2 and 5 |
| Discussion | | |  |  |
| Key results | 18 | Summarise key results with reference to study objectives | page 11-12 line 230-240  page 15, line 319-324 | Discussion Paragraph 1, Paragraph 11 |
| Limitations | 19 | Discuss limitations of the study, taking into account sources of potential bias or imprecision. Discuss both direction and magnitude of any potential bias | page 14, line 308-317 | Discussion Paragraph 10 |
| Interpretation | 20 | Give a cautious overall interpretation of results considering objectives, limitations, multiplicity of analyses, results from similar studies, and other relevant evidence | page 12-15, line 242-306 | Discussion Paragraph 2-9 |
| Generalisability | 21 | Discuss the generalisability (external validity) of the study results | page 14-15, line 308-317 | Discussion Paragraph 10 |
| Other information | | |  |  |
| Funding | 22 | Give the source of funding and the role of the funders for the present study and, if applicable, for the original study on which the present article is based | page 15, line 328 | Funding, Paragraph 1 |

*Give information separately for exposed and unexposed groups.

**Note:** An Explanation and Elaboration article discusses each checklist item and gives methodological background and published examples of transparent reporting. The STROBE checklist is best used in conjunction with this article (freely available on the Web sites of PLoS Medicine at http://www.plosmedicine.org/, Annals of Internal Medicine at http://www.annals.org/, and Epidemiology at http://www.epidem.com/). Information on the STROBE Initiative is available at http://www.strobe-statement.org.
