# Supplementary material for: Mesh-augmented transvaginal repair of recurrent or complex anterior pelvic organ prolapse in accordance with the SCENIHR opinion
Source: Int Urogynecol J. 2020 Sep 24;32(4):819–27. doi: 10.1007/s00192-020-04525-9 (PMC8009781; doi:10.1007/s00192-020-04525-9)
Supplement: Supplementary file 2 — (DOCX 216 kb) [file 192_2020_4525_MOESM2_ESM.docx]

Figure S1 Schematic diagram of crucial steps in Calistar S implantation technique

*A detailed description of the surgical technique can be found in the Calistar S “Surgical Technique Brochure” available* at [www.promedon-urologypf.com/calistar](http://www.promedon-urologypf.com/calistar) *or directly at http://www.promedon-urologypf.com/ecn/uploads/admin/brochures/Surgical_Technique_Brochure_-_Digital_1.pdf*
