# Supplementary material for: Mesh-augmented transvaginal repair of recurrent or complex anterior pelvic organ prolapse in accordance with the SCENIHR opinion
Source: Int Urogynecol J. 2020 Sep 24;32(4):819–27. doi: 10.1007/s00192-020-04525-9 (PMC8009781; doi:10.1007/s00192-020-04525-9)
Supplement: Supplementary file 3 — (DOCX 24.2 kb) [file 192_2020_4525_MOESM3_ESM.docx]

Figure S2 Flow diagram of patient eligibility
