# Supplementary figures and images for: Mesh-augmented transvaginal repair of recurrent or complex anterior pelvic organ prolapse in accordance with the SCENIHR opinion
Source: Int Urogynecol J. 2020 Sep 24;32(4):819–27. doi: 10.1007/s00192-020-04525-9 (PMC8009781; doi:10.1007/s00192-020-04525-9)

Figure S3 Estimated anatomical failure-free survival


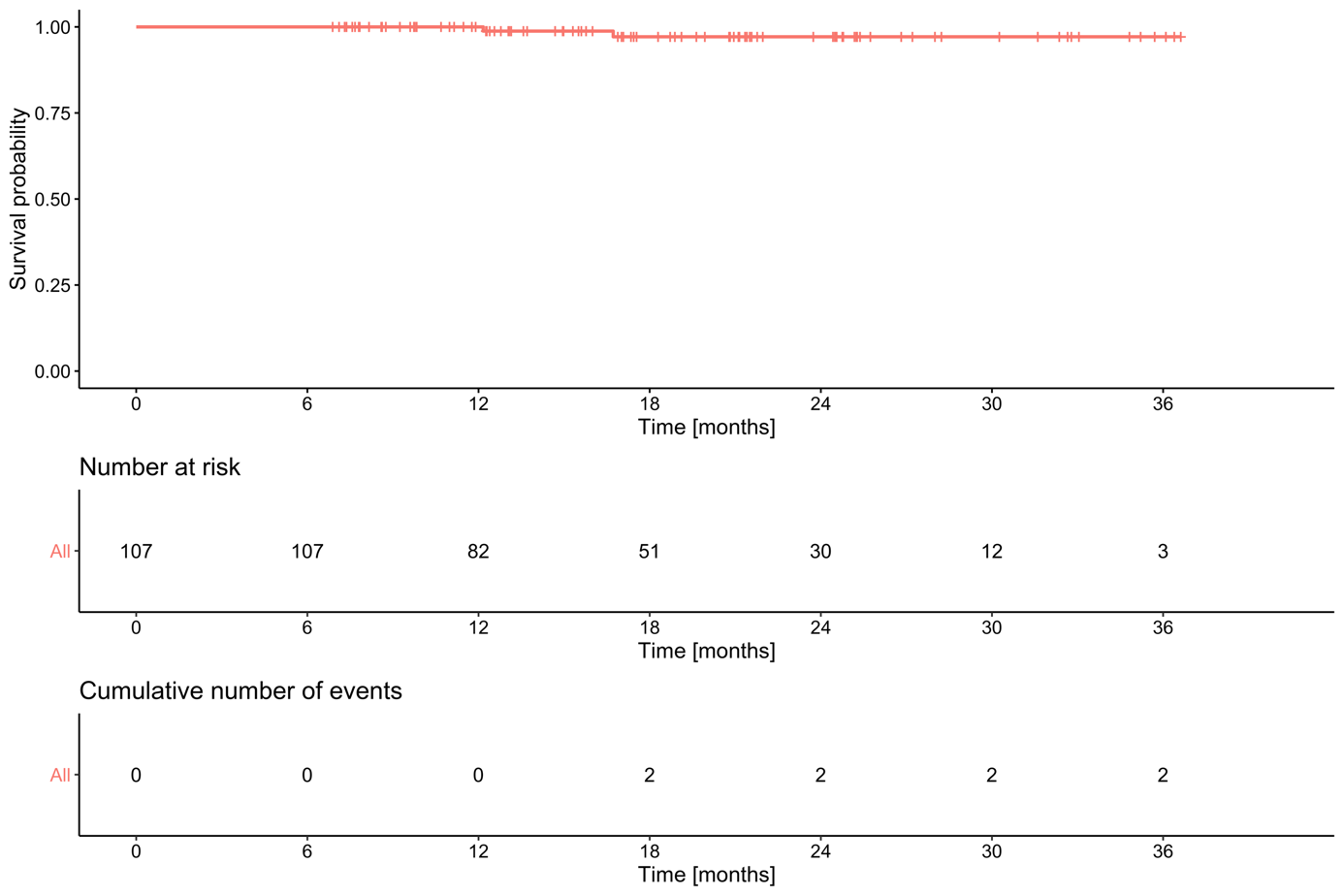

Supplement: Supplementary file 4 — (DOCX 107 kb) [file 192_2020_4525_MOESM4_ESM.docx]

Figure S6 Kaplan-Meier curve for estimated exposure-free survival


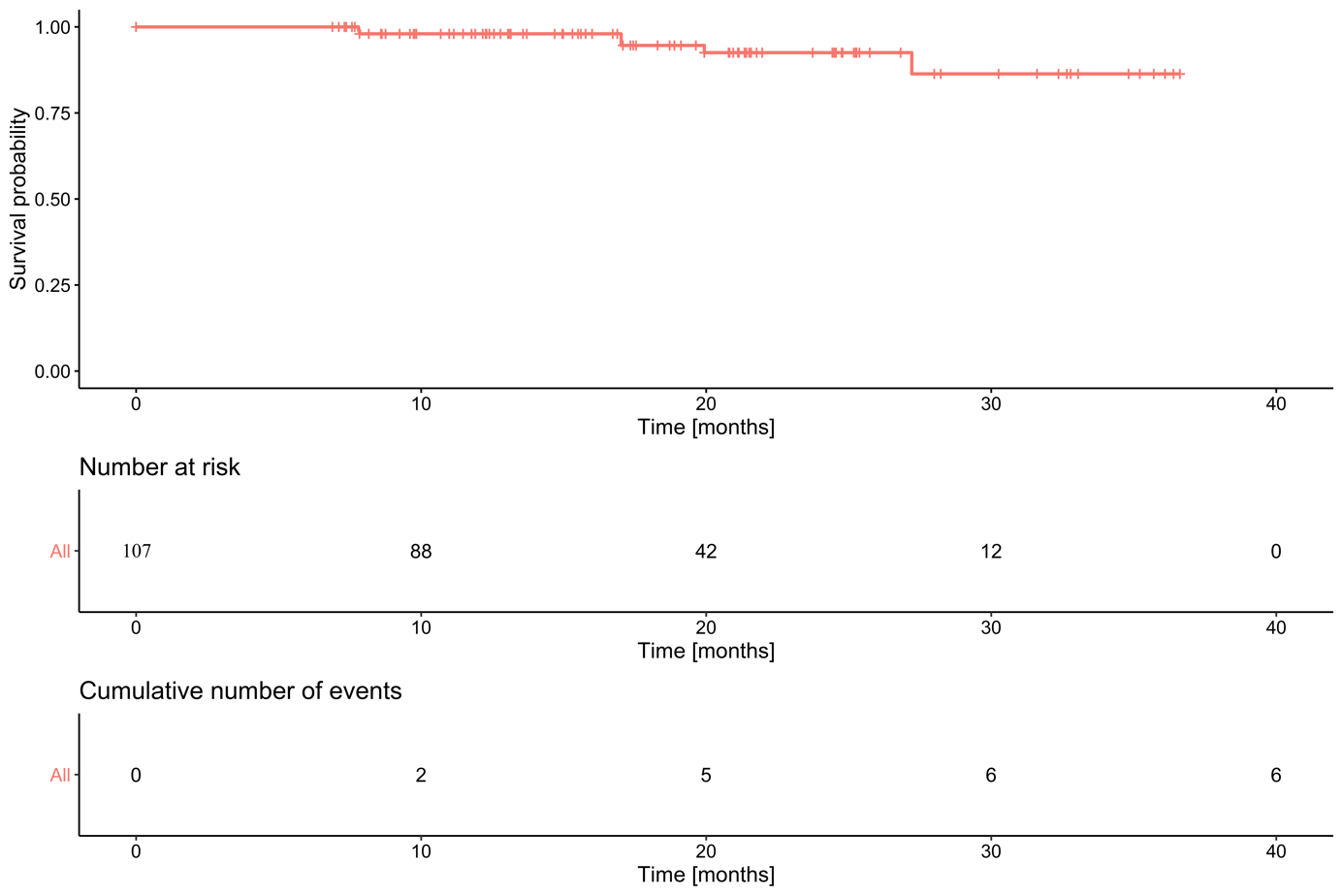

Supplement: Supplementary file 7 — (DOCX 99.1 kb) [file 192_2020_4525_MOESM7_ESM.docx]
