# Supplementary material for: Mesh-augmented transvaginal repair of recurrent or complex anterior pelvic organ prolapse in accordance with the SCENIHR opinion
Source: Int Urogynecol J. 2020 Sep 24;32(4):819–27. doi: 10.1007/s00192-020-04525-9 (PMC8009781; doi:10.1007/s00192-020-04525-9)

Figure S5 Change in the domain”bladder function” of the German Pelvic Organ Prolapse Questionnaire in comparison of Baseline to Follow-Up results


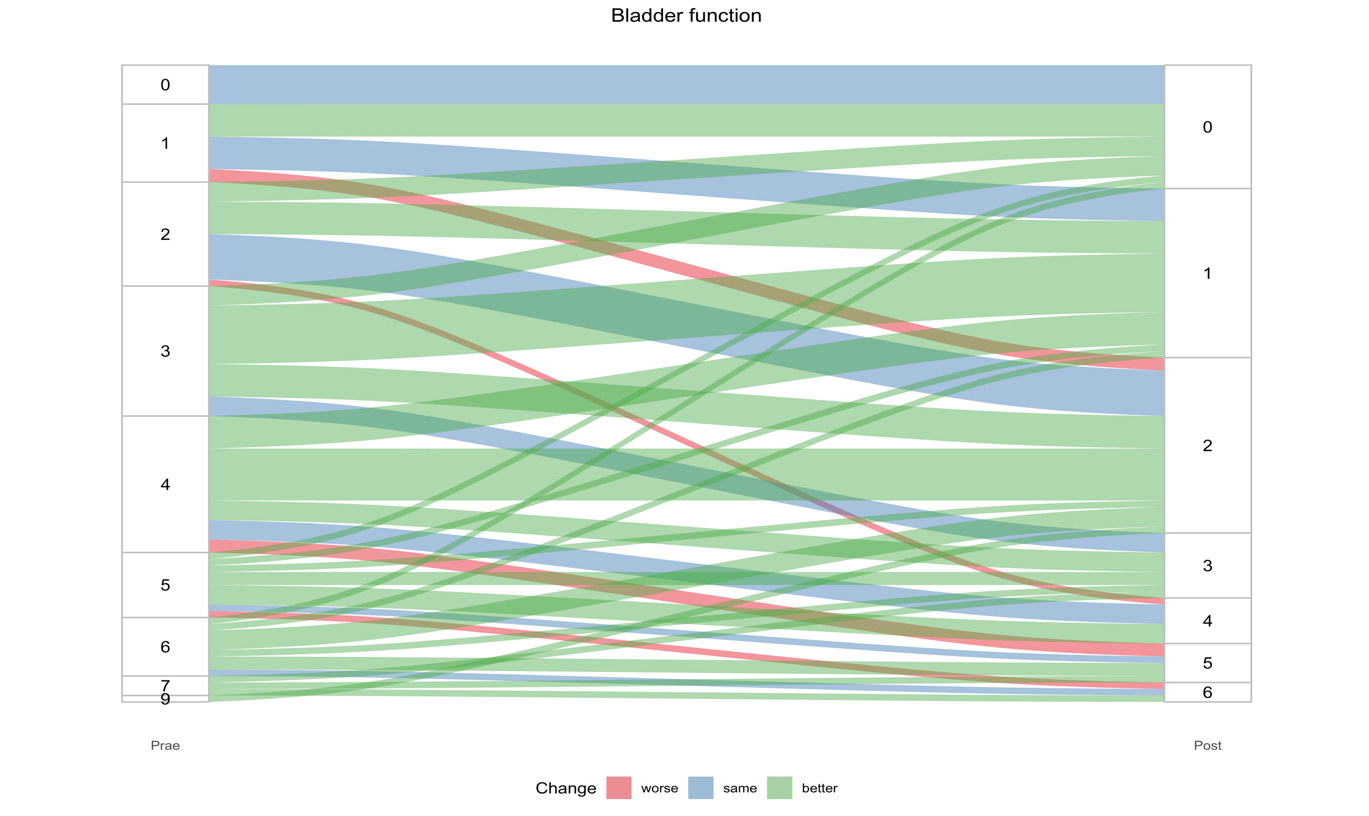

Supplement: Supplementary file 6 — (DOCX 336 kb) [file 192_2020_4525_MOESM6_ESM.docx]
